# Testing How Mindfulness Skills Change for Novice Meditators Using Headspace: Examining Trait Mindfulness and Perceived Stress as Moderators

**DOI:** 10.1002/smi.70215

**Published:** 2026-09-01

**Authors:** Mercedes Peña, Matthew J. Zawadzki, Larisa Gavrilova, Martin S. Hagger

**Affiliations:** ^1^ Department of Psychological Sciences University of California Merced California USA; ^2^ Health Sciences Research Institute University of California Merced California USA

**Keywords:** acceptance, attention, headspace, mHealth, mindfulness, skill learning

## Abstract

Mindfulness‐based interventions are found to effectively reduce stress and improve mental health outcomes. Yet, it is not always clear how the mindfulness skills of attention and acceptance develop throughout the intervention. This knowledge gap is especially pertinent for novice meditators learning these skills for the first time, including whether some individuals are more prone to learning them. Using a randomized waitlist‐controlled trial, we tested the effect of the app Headspace on changes in attention and acceptance over 8 weeks among participants new to mindfulness meditation. Further, we tested the moderating effects of trait mindfulness and perceived stress. Non‐faculty university employees were randomized to a Headspace or waitlist control condition. Trait mindfulness and perceived stress were measured at baseline. Ecological momentary assessment survey data for attention and acceptance were collected five times a day in 4‐day bursts at baseline and 2, 5, and 8 weeks post‐randomisation. Attention and acceptance were significantly higher at Week 8 compared to baseline for the Headspace group, but not the control group. For the Headspace group, both skills showed significant change by Week 2. Trait mindfulness moderated this effect with those who were lower in trait mindfulness displaying greater increases in attention, but not acceptance. Perceived stress also moderated this effect with those who were lower in perceived stress displaying greater increases in attention and acceptance. Our discussion draws attention to implications for matching intervention content to individual needs to ensure participants reporting different levels of characteristics benefit from mindfulness training.

## 1 Introduction

Mindfulness‐based interventions have been found to reduce stress and improve various mental health outcomes (Creswell 2017). These benefits are hypothesised to occur through the learning of mindfulness‐based skills along with continued application to manage stress in daily life (Baer et al. 2012; Carmody and Baer 2008). Preeminent among the skills comprising mindfulness are attention monitoring (i.e., attention skill) and adoption of a nonjudgmental approach to thoughts and feelings (i.e., acceptance skill) (Bishop et al. 2004; Lindsay and Creswell 2017). An important aspect of evaluating mindfulness interventions is whether the intervention leads to changes in the learning of the attention and acceptance skills. Yet, relying on pre‐intervention to post‐intervention evaluation of these skills leaves a gap in understanding as it does not provide sufficient information on *how* mindfulness skills are acquired over the course of an intervention. In the present study we focus on addressing this gap in the literature through examining the effect of Headspace—a smartphone application (app) mindfulness‐based intervention—on trajectories of change in attention and acceptance skill acquisition among a sample of novice meditators. Given that we are interested in novice meditators, we also consider factors that might lead individuals to be more prone to acquisition of these skills. We focus on whether those with higher baseline levels of trait mindfulness—as a proxy for the initial receptiveness (i.e., openness) to mindfulness training—show different patterns of change for each skill than those with lower baseline levels. We also consider the influence of baseline levels of perceived stress on skill acquisition as stress level may influence willingness to engage in the intervention, and it remains unclear whether those who are higher or lower in stress at baseline would show a larger change in each skill.

### 1.1 Skills Underlying Mindfulness‐Based Interventions

Theory on mindfulness has been guided by initial work that points to the importance of attention monitoring and refraining from judgement as distinct skills learnt through mindfulness training that enable individuals to develop the capacity to be mindful. For example, Monitor and Acceptance Theory (MAT) proposes attention and acceptance as the two candidate mechanisms of mindfulness responsible for improved affective and stress reduction outcomes (Lindsay and Creswell 2017), and these mechanisms are presumably built through learning attention and acceptance skills. According to MAT, the skill of *attention* enhances awareness of affective information, while *acceptance* encourages nonjudgement and openness towards the affective information gathered when practicing attention (Lindsay and Creswell 2017). Similarly, Bishop et al. (2004) indicate that the maintenance of a ‘mindful state’ is dependent on the regulation of attention and cultivation of an open orientation to experiences. The recognition of attention and acceptance as the key components underlying mindfulness suggests development of these skills can potentially be used as an early indicator of whether a novice meditator is learning how to be mindful when participating in a mindfulness‐based intervention, but this relies on having a picture of the trajectories of the skills during the intervention period.

### 1.2 Trajectories of Mindfulness Skill Acquisition

As noted, engagement in a mindfulness intervention prompts practice of attention monitoring and nonjudgement, but the rate or trajectory at which the attention and acceptance skills are acquired may be different, such as indicated in a previous study (i.e., Baer et al. 2012). Importantly, models of skill acquisition propose that skill training facilitates progressive, incremental change in skill acquisition and proficiency over time until the skill is fully acquired (Anderson 1982; Hagger et al. 2010). When practicing mindfulness, the rate of change for acquiring the attention skill may be shorter than for the acceptance skill given that attention monitoring may be necessary prior to practicing acceptance (Lindsay and Creswell 2017). It may also be the case that attention is an easier skill to learn and thus acquired at a quicker rate, while acceptance is more difficult to learn and thus acquired at a slower rate; however, there is no clear evidence to support this idea. Overall, these points might indicate that during the course of a mindfulness intervention, an initial change in the attention skill is likely to be observed prior to a change in the acceptance skill. Consistent with this premise, prior work has indicated statistically significant change in the attention subscale of a mindfulness measure from week 1 to week 2 and was sustained until week 7, the final week of the intervention (Baer et al. 2012). While for the nonjudgement subscale significant change was observed from week 1 to week 3 and then again at week 7 (Baer et al. 2012). In other work on novice meditators, downstream benefits from presumed changes in mindfulness were also observed at different time points. Specifically, there were increases in nonreactivity and decentering within 2 weeks of training but no changes to self‐compassion (Gavrilova and Zawadzki 2023). This suggests people may learn different mindfulness skills, including attention and acceptance, at different rates.

### 1.3 Moderators of Mindfulness Skill Acquisition: Trait Mindfulness and Perceived Stress

Many individual characteristic moderators, including neuroticism (Nyklíček and Irrmischer 2017), conscientiousness (de Vibe et al. 2013), and baseline mindfulness (Shapiro et al. 2011), have been considered in investigations of the effects of MBIs on outcomes. These studies generally find that personality factors and mindfulness moderate the effect of these interventions on distress‐related outcomes. Importantly, these investigations have primarily focused on testing these factors as moderators of the effects of MBIs on treatment outcomes, such as stress reduction and higher well‐being. Thus, there remains a need to consider the effect of moderators on change in key skills, and particularly among people who are new to practicing. More specifically, individual characteristics like trait mindfulness may influence mindfulness skill acquisition, such that some people are able to learn these skills more effectively during participation.

Trait mindfulness refers to holding a steady mindful disposition in daily life (Baer et al. 2006). Individuals higher in trait mindfulness are said to be more mindful across different situations and contexts, and these individuals may perceive being mindful as a core component of their identity. Trait mindfulness has been found to hold relationships with self‐reported executive function and emotion regulation, such that higher mindfulness is associated with greater mood regulation and executive function (Lyvers et al. 2014). Similarly, increased trait mindfulness has been associated with increased emotion regulation, openness, and acceptance (Polizzi et al. 2018). More recent research indicates an association between trait mindfulness awareness and state mindfulness dimensions during meditation practice (Brahmi et al. 2025). Thus, individual differences in trait mindfulness prior to participation in a mindfulness‐based intervention may make some individuals more adept at skill acquisition. Particularly, novice meditators high in trait mindfulness may naturally possess a ‘mindful mindset’ that results in unintentional practice and maintenance of attention and acceptance skills, making learning these skills during the intervention easier. Trait mindfulness potentially supports engagement in the practices and learning of the key skills. For example, in a study of undergraduate participants, those with higher pre‐treatment levels of mindfulness showed a greater increase in overall mindfulness levels at post‐treatment after participating in a mindfulness intervention (Shapiro et al. 2011). An extension of this previous work is needed to test whether trait mindfulness is directly connected to how people, more specifically novices, learn mindfulness skills when participating in an intervention.

Similarly, novice meditators' initial levels of perceived stress may influence mindfulness skill acquisition. Perceived stress refers to the degree to which situations in life are appraised as stressful (Cohen et al. 1983). Individuals who are higher in perceived stress at baseline may engage in mindfulness practice in an attempt to address feelings of stress, and engagement may in turn contribute to greater change in attention and acceptance skills over the intervention period. Research on stress as a moderator of the effect of mindfulness‐based interventions on mindfulness skill learning and treatment outcomes is limited. However, perceived stress has previously been found to moderate the effect of a mindfulness acceptance induction on the treatment outcome of coping (Donald and Atkins 2016). More specifically, the mindfulness induction produced more approach and less avoidance coping among participants reporting high levels of perceived stress at baseline. Somewhat related, one study testing a general web‐based stress management intervention found that higher distress at baseline moderated intervention effectiveness (Coudray et al. 2019). Alternatively, in general domains of learning it is found that high levels of baseline perceived stress *negatively* impact learning (Oducado and Estoque 2021). Thus, it remains necessary to test baseline perceived stress as a moderator to clarify whether higher perceived stress influences learning of attention and acceptance skills during mindfulness mediation intervention participation and in what manner (i.e., lesser or greater change). This is especially important given that mindfulness meditation is often suggested as an appropriate and useful tool to address heightened stress via increases in mindfulness (i.e., greater attention and acceptance).

### 1.4 App‐Based Mindfulness Meditation as an Intervention

Although delivery of mindfulness based interventions has typically occurred in clinical or in‐person contexts in which a trained clinician or facilitator works with individuals or groups in the provision of mindfulness skill training, there is increasing evidence that these interventions can be delivered online or through internet‐enabled smartphone ‘apps’ without considerable loss in fidelity or outcomes (Mani et al. 2015). Mindfulness meditation apps can be useful to allay concerns cited as barriers to uptake and adherence of in‐person interventions, including cost, time commitment, access, and reach (Goldberg et al. 2020). A prominent example of a mindfulness‐based app often used as an intervention in research contexts is Headspace (Headspace, Santa Monica, CA, USA), which has demonstrated widespread popularity and provides access to a variety of guided meditation practices. Research indicates that mindfulness training delivered via apps are efficacious in increasing mindfulness and improving mental health outcomes (Gál et al. 2021), including in showing reductions to various types of stress in the current sample (Zawadzki et al. 2025). However, evidence of how these apps facilitate the learning of key mindfulness skills is scarce by comparison. Practicing with Headspace provides the opportunity to participate in mindfulness meditation throughout the day at the convenience of the user, but there remains a need to test change in attention and acceptance skills over the course of using the app, especially among novice meditators.

### 1.5 Assessing Key Mindfulness Skills in Everyday Life

Initial investigations measuring mindfulness outcomes were focused on using between‐group designs testing change in mindfulness measures from pre‐intervention to post‐intervention, but we now recognise it is important to understand the structure of that change. One way to examine the extent to which people are mindful in daily life throughout a variety of changing situations is through a randomized control design with multiple assessments of attention and acceptance. Ecological momentary assessment (EMA) can facilitate achieving this goal. EMA is a measurement method in which participants are prompted to provide data at an intensive frequency (Shiffman et al. 2008). This approach enables greater data fidelity by reducing measurement error arising from recall or other artefacts. Use of this measurement method provides the opportunity to appropriately examine the trajectory of each mindfulness skill. It also allows for testing variation in the trajectories of the skills to examine potential intraindividual conditions that may moderate the effects. Thus, EMA can be used to uniquely measure the embeddedness of learning mindfulness in daily life by measuring change in the skills of attention and acceptance over varying contexts during an app‐based mindfulness intervention.

### 1.6 The Present Study

In the current study we conducted an analysis of a mindfulness skill training intervention adopting a randomized, waitlist‐controlled design. We aimed to extend prior work that examined how training in mindfulness affected cognitive mechanisms (i.e., decentering, self‐compassion, and mindfulness factor) (Gavrilova and Zawadzki 2023). This prior work focused on assessment of these cognitive mechanisms in terms of how they were applied in everyday life. We use novel data, analysing different items aiming to measure the development of mindfulness in terms of how aware and open a person views themselves in a moment, thus focusing more on the inward facing processes. These data allow focus on the acquisition of attention and acceptance skills during mindfulness training and pull apart the process by which novice meditators become mindful. We also extend this prior work by considering who has a greater change in these skills. Specifically, we test the moderating effects of trait mindfulness and perceived stress, person‐level factors that might affect this process and were not considered in the previous study, on mindfulness skill acquisition.

We expected attention and acceptance would increase over the course of the intervention in the mindfulness meditation group as compared to the waitlist control group (H1). We hypothesised that individuals randomized to the mindfulness meditation group would exhibit an increase in attention with expected changes happening rapidly—by Week 2 (H2a). We also hypothesised that individuals randomized to the mindfulness group would exhibit an increase in acceptance, but with expected changes occurring more slowly—by Week 5 (H2b). We predicted that individuals randomized to the mindfulness group who were higher (vs. lower) on trait mindfulness would show larger increases in attention and acceptance (H3). Finally, we formulated competing hypotheses for moderation tests of perceived stress. We predicted that individuals randomized to the mindfulness group who were lower (vs. higher) in perceived stress would show larger increases in attention and acceptance (H4a). We also predicted that individuals randomized to the mindfulness group who were higher (vs. lower) in perceived stress would show larger increases in attention and acceptance (H4b). The value of the current analyses is that researchers will be provided with novel information on the development of mindfulness skills through identification of trajectories for the specific underlying skills and consideration of factors that might affect those trajectories, which begins to address a current gap in the literature regarding skill acquisition in mindfulness interventions. This will also add to the current knowledge on the efficacy of mindfulness apps to change the key underlying skills of attention and acceptance in the expected direction (i.e., increase these skills), which is important given their growing use.

## 2 Method

### 2.1 Participant Sample

Participants were non‐faculty staff at a public university in Central California. Eligible employees were recruited through emails and flyers advertising a study testing the effects of mindfulness meditation on workplace stress. Those expressing an interest in participating completed an initial online screener survey to assess eligibility. Inclusion criteria were access to a smartphone, consistent access to internet, fluency in English, non‐student and non‐faculty employee status, and aged 18 years or older. People who were experienced meditators, defined here as those who participated in sitting meditation more than twice a week for 10 minutes or more over the previous 3 months, were also excluded from participating.

**FIGURE 1:**
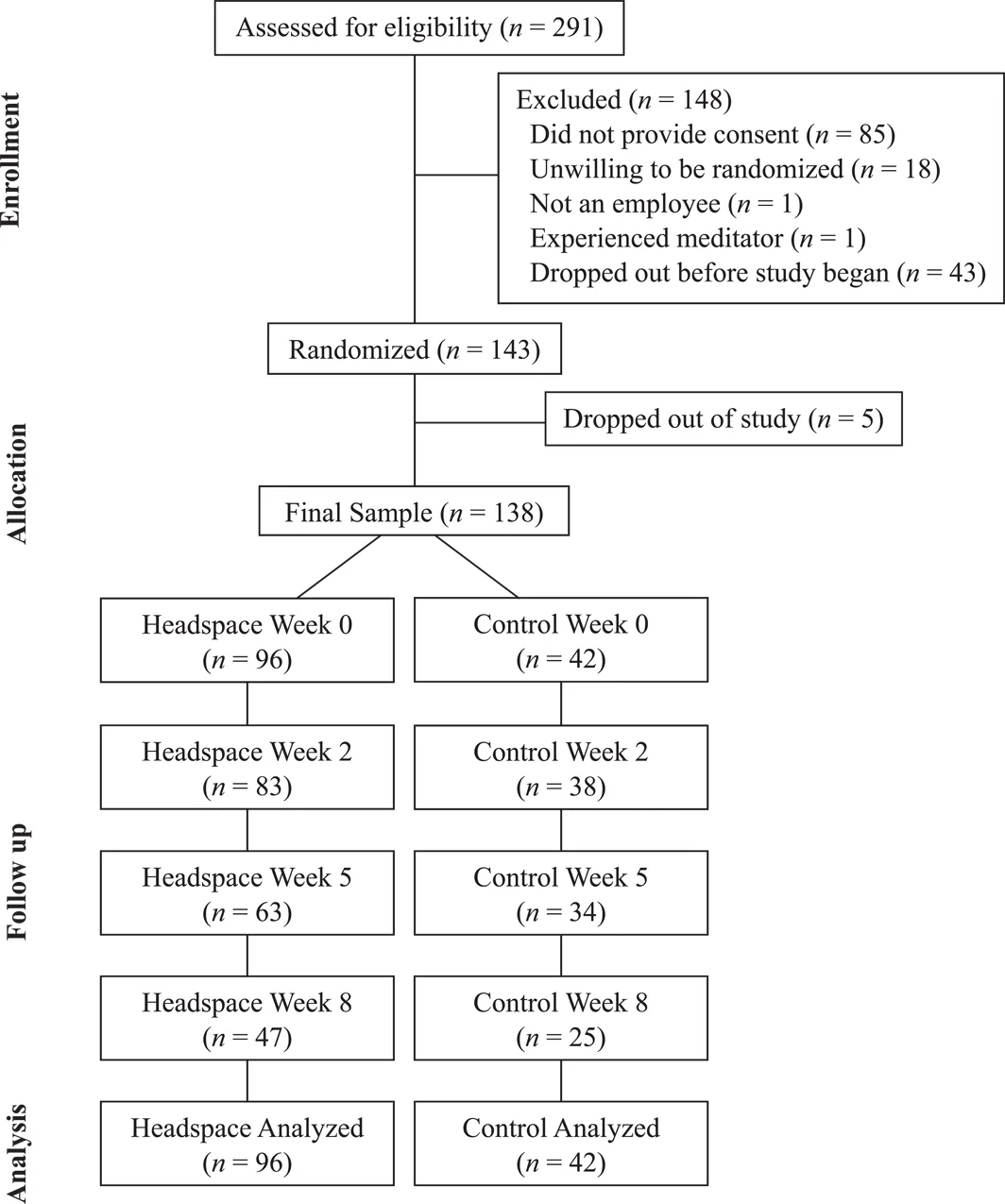
CONSORT diagram.

To determine the appropriate sample size, a power analysis was conducted. Given prior research indicating a moderate effect could be observed with this form of mHealth app (Baumel et al. 2019), a moderate effect size, an alpha of 0.05, and a power of 0.80 were assumed in the power analysis. The analysis indicated a sample size of 128 participants would be needed to detect an effect leading to the aim to enrol 140 participants to account for possible attrition. From the individuals initially screened for eligibility (*N* = 291), 20 were ineligible as they were a student or faculty employee. Of the remaining employees eligible for participation, 186 provided consent. However, prior to the start of the study 43 participants dropped out leaving 143 eligible participants for randomisation. Following randomisation, five participants dropped out resulting in a final sample of 138 participants for the analyses (see Figure 1 for flow of participants).

### 2.2 Procedure

At baseline, after completing an informed consent form, eligible participants received a link to a Qualtrics survey comprising measures of participants' demographic characteristics (i.e., age, gender, race/ethnicity), trait mindfulness, and perceived stress. Once completed, participants were required to attend an initial 60‐min in‐person orientation session facilitated by a trained researcher. In the session, participants were guided to download the LifeData app (RealLife Exp, Life Data Corporation, Marion, IN) on their smartphone and instructed on how to navigate the app. This training included answering practice questions to familiarise themselves with the process of responding to EMA surveys throughout the study. At the end of the in‐person orientation session, participants were provided with a printed user guide containing information about each of the EMA questions, frequency of the surveys, other information relevant to the study, FAQs related to the LifeData app, and researcher contact information.

Following completion of the baseline survey and the training session, participants were randomized to either an intervention condition in which they received mindfulness delivered via Headspace or to a waitlist control condition for 8 weeks using a 2:1 allocation procedure. Participants in the intervention group were sent an email containing instructions for downloading the Headspace app and a code providing free access to materials for 12 months. Headspace is a commercially available mindfulness meditation app (https://www.headspace.com) providing guided and unguided training aligned with traditional in‐person mindfulness practices. The app has been used in prior intervention research (Flett et al. 2020) and been rated as high‐quality in comparison to other commercially available meditation apps (Mani et al. 2015). In Headspace, instructions for meditation are delivered via short, animated videos and sound files.

Participants allocated to the intervention group were instructed to meditate for 10 minutes a day for 8 weeks using the app. This group began using the Basics pack in Headspace, which is designed to introduce the user to mindfulness by teaching practices related to both attention and acceptance. Following the completion of Basics 1, 2, and 3, each consisting of 10 sessions approximately 10 minutes in length, participants completed the Stress pack. This consists of 30 sessions each 10 minutes in length and has content more targeted towards stress. App downloading and initial use was tracked for the intervention group, and those who did not download or use the app within the first week received text message reminders. Those allocated to the waitlist control group were asked to refrain from any meditation exercises and received similar access to the app after 4 months.

EMA survey data related to mindfulness skills were collected using the LifeData app in 4‐day bursts at baseline (Week 0), and 2, 5, and 8 weeks post‐randomisation. Participants received the surveys at random times within specified timeframes—8:00am–10:00am, 10:30am–12:00pm, 1:00pm–3:00pm, 3:30pm–5:30pm, 6:00pm–8:00pm—five times a day for four consecutive days (i.e., Wednesday through Saturday) during each burst. Participants were remunerated $15 for each burst they completed and an additional $20 remuneration for a high completion rate (i.e., 80% of EMA surveys completed).

### 2.3 Measures

#### 2.3.1 Baseline Moderator Assessment

Trait mindfulness was measured using the Mindful Attention Awareness Scale (MAAS; Brown and Ryan 2003). The MAAS is a 15‐item scale designed to assess the core characteristic of mindfulness with the majority of questions asking about attention across moments or situations. Example items from the MAAS include, ‘I tend to not notice feelings of physical tension or discomfort until they really grab my attention’ and ‘I rush through activities without being really attentive to them’. Participants were asked to reflect on their everyday experiences and then indicate how frequently or infrequently they experienced each statement on a scale from 1 (*almost always*) to 6 (*almost never*). The MAAS has been validated in previous work (MacKillop and Anderson 2007), and reliability for the present sample was good (*α* = 0.89).

Stress was measured using the Perceived Stress Scale (PSS; Cohen et al. 1983). The PSS is a 10‐item scale designed to measure perceptions of stress over the past month. Example items from the PSS include, ‘In the last month, how often have you felt that you were unable to control the important things in your life’ and ‘In the last month, how often have you felt difficulties were piling up so high that you could not overcome them’. Participants were asked to indicate how often they felt or thought in a certain way by rating each statement on a scale from 0 (*never*) to 4 (*very often*). The reliability for the present sample was good (*α* = 0.87).

#### 2.3.2 EMA Skill Assessment

To assess the key mindfulness skills of attention and acceptance at a momentary level, we adapted items from commonly used scales (Bishop et al. 2004; Brown and Ryan 2003), an approach employed in previous work (i.e., Blanke and Brose 2017). To measure the skill of attention, the item ‘At this moment, I feel attentive to and aware of the present moment’ was used. For the skill of acceptance, the item ‘At this moment, I experience the attitude of openness and acceptance’ was used. Both items were rated on a scale from 0 (*not at all*) to 10 (*extremely*). These items were presented in the same order each time for all participants, with the attention item asked before the acceptance item.

### 2.4 Analytic Plan

Chi‐square and *t*‐tests were used to assess group differences in baseline characteristics, trait mindfulness, and perceived stress. Age, gender, and ethnicity were included as control variables as stated in the pre‐registration, and also included were study day, time of day, and type of day (i.e., weekday vs. weekend) to account for shifts that will naturally occur over time rather than due to the intervention.

Hypothesis 1 Focuses on the change in the skills of attention and acceptance across baseline, Week 2, Week 5, and Week 8. Given that the data had a multilevel structure with weeks (Level 1) nested within participants (Level 2), multilevel mixed effect modelling was used. Week (0, 2, 5, 8) and study condition (i.e., intervention or waitlist control) and the interaction between these were entered as predictors. A single weekly score was calculated for each participant using their available weekly data to obtain an average score of each skill for that week. Participants could have up to 20 observations for any given week and had to have at least five observations to be considered reliable and not deemed missing. We tested whether attention and acceptance were significantly different in the intervention group as compared to the control group at Week 8.

Hypothesis 2 Focuses on the rate of change of attention and acceptance across time for those in the Headspace group. Weeks (0, 2, 5, 8) were entered as the predictor and all other modelling was the same as for testing Hypothesis 1. Skills at Weeks 2 and 5 were compared to baseline for the intervention group and control group to test whether the skill of attention changed earlier than the skill of acceptance, for example at Week 2 versus Week 5.

Hypothesis 3 Focuses on the change in attention and acceptance among those high in trait mindfulness at baseline as compared to those low in trait mindfulness at baseline. The same models presented for testing Hypothesis 1 were run, with the inclusion of an interaction between Week and trait mindfulness. Trait mindfulness was grand mean centred. We tested whether increases in these mindfulness skills are larger among those high in trait mindfulness as compared to those low in trait mindfulness. To visualise these interaction effects, we used the interActive tool (McCabe et al. 2018). This allowed us to generate figures displaying the relationship between time (in weeks) and attention at varying levels of trait mindfulness for each group while controlling for the variables mentioned above.

Hypothesis 4 Focuses on the change in attention and acceptance as a result of the interaction between being in the Headspace group, Week, and baseline perceived stress. The same models presented for testing Hypothesis 3 were run; however, the interaction was between Week and perceived stress. Perceived stress was grand mean centred. We tested whether mindfulness skill increases differed according to baseline level of perceived stress. We used the interActive tool (McCabe et al. 2018) to visualise these interaction effects, allowing us to display the relationship between time and each skill at varying levels of perceived stress. All analyses were conducted using SAS version 9.4 and pseudo *R* ^2^ values are used as a measure of effect size for each model given the multilevel structure of the data. These values were calculated by outputting a score for attention/acceptance at each moment based on model parameters and observed values, and then correlating this output score with the actual attention/acceptance score.

## 3 Results

### 3.1 Descriptive Analyses

Descriptive statistics for participant characteristics are found in Table 1. At baseline, prior to the intervention, participants assigned to the control condition (*M* = 41.12, SD = 10.71) were slightly older in age compared to participants in the Headspace condition (*M* = 36.91, SD = 10.84), *t*(136) = 2.11, *p* = 0.037. Those allocated to the control group did not significantly differ from those in the Headspace group on baseline mindfulness (*M* = 3.61, SD = 0.85 vs. *M* = 3.69, SD = 0.87, respectively; *t*(136) = −0.48, *p* = 0.63) and baseline stress (*M* = 18.07, SD = 5.36 vs. *M* = 19.56, SD = 5.93, respectively; *t*(136) = −1.39, *p* = 0.17). Individuals assigned to the Headspace and control groups also did not significantly differ on the proportion identifying as Hispanic (31.25% vs. 19.05%, respectively; *χ* ^2^ = 2.18, *p* = 0.14) or identifying as female (77.08% vs. 71.43%, respectively; *χ* ^2^ = 0.50, *p* = 0.48).

**TABLE 1:** Descriptive statistics for demographic characteristics, trait mindfulness, and perceived stress at baseline (week 0).

	Intervention (*n* = 96)	Waitlist‐control (*n* = 42)	Total (*n* = 138)
Age			
Mean (SD)	36.91 (10.84)	41.12 (10.71)	38.18 (10.94)
Gender (*n*, %)			
Female	74, 77.08%	30, 71.43%	104, 75.36%
Male	22, 22.92%	12, 28.57%	34, 24.64%
Ethnicity (*n*, %)			
Non‐hispanic	66, 68.75%	34, 80.95%	100, 72.46%
Hispanic	30, 31.25%	8, 19.05%	38, 27.54%
Trait mindfulness			
Mean (SD)	3.69 (0.87)	3.61 (0.85)	3.66 (0.86)
Perceived stress			
Mean (SD)	19.56 (5.93)	18.07 (5.36)	19.11 (5.78)

When comparing completers (i.e., those present for the entire intervention period and non‐completers) (i.e., those who dropped out at some point in the intervention period), those who stayed (*M* = 38.43, SD = 11.43) were similar in age to those who dropped out (*M* = 37.92, SD = 10.46, *t*(136) = −0.27, *p* = 0.79. Completers also did not differ from non‐completers on baseline mindfulness *M* = 3.67, SD = 0.81 versus *M* = 3.66, SD = 0.91, respectively; *t*(136) = −0.03, *p* = 0.98) or baseline stress (*M* = 19.10, SD = 5.74 vs. *M* = 19.11, SD = 5.87, respectively; *t*(136) = 0.02, *p* = 0.99). Finally, those who completed the intervention and those who did not complete the intervention did not significantly differ on the proportion identifying as Hispanic (26.39% vs. 28.79%; *χ* ^2^ = 0.10, *p* = 0.75) or identifying as female (81.94% vs. 68.18%, respectively; *χ* ^2^ = 3.51, *p* = 0.06).

In further examining whether attrition patterns differ by condition, we found that completion did not significantly differ by condition (48.96% in Headspace vs. 59.52% in control; *χ* ^2^ = 1.31, *p* = 0.25). When considering differences within conditions, the Headspace completers and non‐completers did not significantly differ on the proportion identifying as Hispanic (31.91% vs. 30.61%, respectively; *χ* ^2^ = 0.02, *p* = 0.89) or identifying as female (85.11% vs. 69.39%, respectively; *χ* ^2^ = 3.35, *p* = 0.07). Completers in the Headspace group were also similar in age (*M* = 37.23, SD = 11.39) to non‐completers (*M* = 36.59, SD = 10.39), *t*(94) = −0.29, *p* = 0.77. Headspace completers also did not differ from non‐completers on baseline mindfulness (*M* = 3.60, SD = 0.82 vs. *M* = 3.77, SD = 0.91, respectively; *t*(94) = 0.96, *p* = 0.34) or baseline stress (*M* = 20.23, SD = 6.04 vs. *M* = 18.91, SD = 5.81, respectively; *t*(94) = −1.10, *p* = 0.28). Similarly, control group completers and non‐completers did not differ on the proportion identifying as Hispanic (16.00% vs. 23.53%, respectively; *χ* ^2^ = 0.37, *p* = 0.54) or identifying as female (76.00% vs. 64.71%, respectively; *χ* ^2^ = 0.63, *p* = 0.43). Control completers were also similar in age (*M* = 40.68, SD = 11.38) to non‐completers (*M* = 41.76, SD = 9.95), *t*(40) = 0.32, *p* = 0.75. Control completers also did not differ from non‐completers on baseline mindfulness (*M* = 3.79, SD = 0.80 vs. *M* = 3.35, SD = 0.87, respectively; *t*(40) = −1.70, *p* = 0.10) or baseline stress (*M* = 16.96, SD = 4.51 vs. *M* = 19.71, SD = 6.18, respectively; *t*(40) = 1.67, *p* = 0.10).

### 3.2 Headspace by Week Analyses

The same interaction effect between intervention group by time was used to test hypotheses 1 and 2, with different planned comparisons being used for each hypothesis. To address Hypothesis 1, we tested whether attention and acceptance were significantly different in the intervention group and control group at Week 8 as compared to Week 0 (baseline). Descriptive statistics for levels of attention and acceptance by week and condition can be found in Table 2. As seen in Table 3 and confirming our hypothesis, those in the Headspace group compared to the control group reported more attention at Week 8 compared to baseline (*t*(6115) = 2.73, *p* = 0.006), whereas the control group reported no difference compared to baseline (*t*(6115) = −1.22, *p* = 0.22). The Headspace group compared to the control group also reported more acceptance at Week 8 compared to baseline (*t*(6100) = 3.72, *p* < 0.001), while the control group reported no difference compared to baseline (*t*(6100) = −1.14, *p* = 0.26).

**TABLE 2:** Means (standard deviations) for attention and acceptance across weeks by condition.

	Attention			Acceptance		
	Headspace	Control	Total	Headspace	Control	Total
Week 0	6.30 (2.06)	6.45 (1.97)	6.35 (2.03)	6.24 (2.09)	6.38 (2.09)	6.28 (2.09)
Week 2	6.34 (1.95)	6.18 (1.79)	6.29 (1.90)	6.41 (1.96)	6.11 (1.90)	6.31 (1.95)
Week 5	6.73 (2.05)	6.21 (1.95)	6.56 (2.03)	6.74 (1.97)	6.24 (1.94)	6.58 (1.97)
Week 8	6.66 (2.15)	6.50 (1.83)	6.60 (2.04)	6.64 (2.21)	6.38 (1.94)	6.54 (2.12)

*Note:* Week 0 is baseline.

**TABLE 3:** Interactive effects of condition and time (in weeks) on attention and acceptance.

	Attention	Acceptance
	*b* (SE)	*b* (SE)
Random Effects		
Intercept	1.55* (0.20)	1.56* (0.20)
Residual	2.37* (0.04)	2.43* (0.04)
Fixed Effects		
Intercept	5.45* (0.54)	5.62* (0.54)
Female	0.13 (0.25)	0.16 (0.25)
Age	0.02 (0.01)	0.01 (0.01)
Hispanic	−0.02 (0.26)	0.06 (0.26)
Weekend	0.25* (0.05)	0.28* (0.05)
Study day	0.03* (0.02)	0.04* (0.02)
Time of day	0.00 (0.01)	0.01 (0.01)
Week 2	−0.28* (0.09)	−0.28* (0.09)
Week 5	−0.34* (0.10)	−0.29* (0.10)
Week 8	−0.13 (0.11)	−0.13 (0.11)
Headspace	−0.16 (0.25)	−0.21 (0.25)
Headspace × week 2	0.35* (0.11)	0.53* (0.11)
Headspace × week 5	0.67* (0.12)	0.79* (0.12)
Headspace × week 8	0.37* (0.14)	0.51* (0.14)
Model Statistics		
Pseudo *R* ^2^	0.02	0.02

*Note:* Weeks 2, 5, and 8 are compared to Week 0 (baseline). Gender, Age, and Ethnicity are demographic variables and standard controls in previous tests of mindfulness‐based interventions. Weekend, Study Day, and Time of Day are standard controls in EMA studies to account for shifts that will naturally occur over time rather than due to the intervention.

Abbreviations: *b* = unstandardised beta coefficient; SE = standard error of unstandardised beta coefficient.

^*^ *p* < 0.05.

To address hypothesis 2, we focused on the Headspace group only. We tested whether the rate of change for each skill was different over the 8‐week intervention, comparing each week to baseline. First, as expected, attention increased by Week 2 compared to baseline (*t*(6115) = 3.15, *p* = 0.002), and continued showing this increase at Week 5 (*t*(6115) = 5.54, *p* < 0.001) and Week 8 (*t*(6115) = 2.73, *p* = 0.006). Second, we unexpectedly saw acceptance also increase by Week 2 compared to baseline (*t*(6100) = 4.70, *p* < 0.001), with this increase present at Week 5 (*t*(6100) = 6.49, *p* < 0.001) and Week 8 (*t*(6100) = 3.72, *p* < 0.001) as well.

To further examine the results for hypothesis 2, we conducted follow‐up analyses. In these analyses, the same models described above were used, however they only included data from Weeks 2 and 5 or Weeks 5 and 8. These comparisons tested whether attention and acceptance continued to change after Week 2 and unstandardised estimates are reported here. For attention there was a significant increase from Week 2–5 (*b* = 0.34, *t*(4043) = 2.85, *p* = 0.004). Attention decreased, however, from Week 5 to Week 8 (*b* = −0.49, *t*(2207) = −3.32, *p* = 0.001), though it was still greater than at baseline. For acceptance there was only a marginal increase from Week 2–5 (*b* = 0.23, *t*(4031) = 1.93, *p* = 0.054), and a decrease from Week 5–8 (*b* = −0.46, *t*(2200) = −3.22, *p* = 0.001), though it remained above baseline.

### 3.3 Trait Mindfulness Moderation Analyses

**FIGURE 2:**
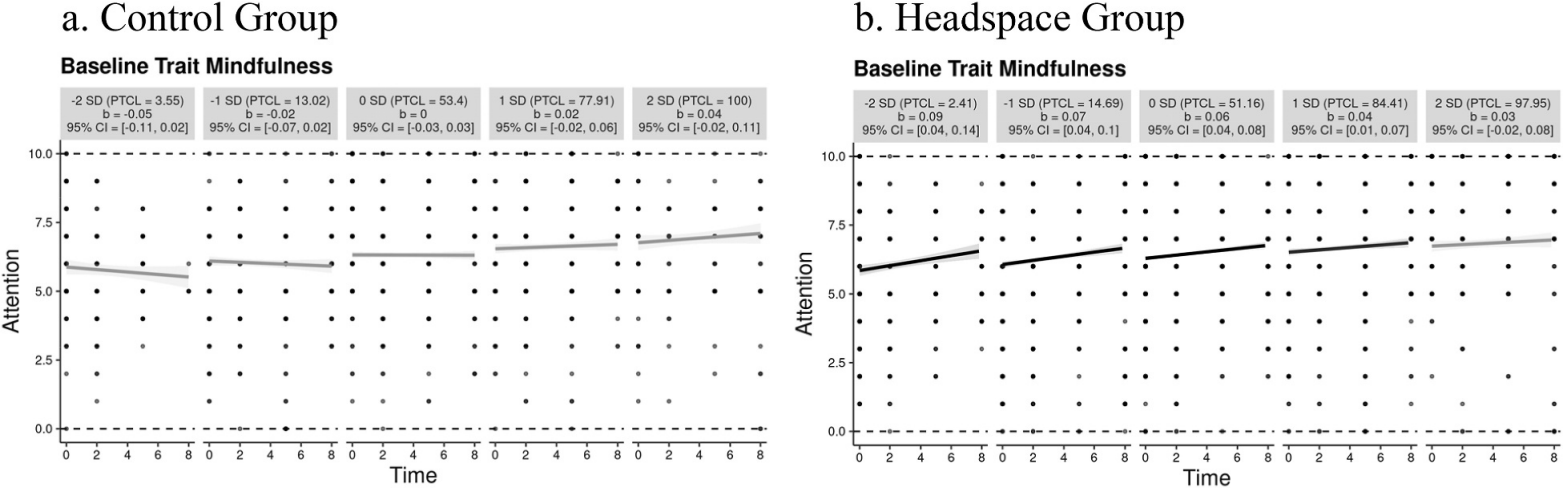
Levels of attention as a function of time (in weeks) and trait mindfulness for the control (Panel 2a) and headspace (Panel 2b) conditions. (Panel 2a) Control group. (Panel 2a) Headspace group. Figure depicts the relationship between time (in weeks) and attention. (Panel A) presents the data for the control group and Panel B for the Headspace group. Within each panel, each figure is separated into five sub‐panels to visualise the effect of trait mindfulness as a moderator. These sub‐panels present the simple slopes for different levels of the moderator, including 2 and 1 SD below the mean, at the mean, and 1 and 2 SD above the mean. Each box above the sub‐panel graph indicates the estimated beta coefficient between time (in weeks) and attention, as well as the 95% confidence interval for the effect. Each sub‐panel displays a graph of the observed data points plotted (black dots), the 95% confidence region (shaded area) around the simple slope, and the maximum and minimum values of the outcome (dashed horizontal lines). Overall, the figures show that for the control group (Panel A), there is little change over the 8 weeks regardless of the levels of trait mindfulness. In contrast, for the Headspace group (Panel B), there are significant increases in attention for all levels of trait mindfulness, except the highest level, with steeper increases among those with lower trait mindfulness. CI = confidence interval; *PTCL* = percentile; SD = standard deviation.

To address hypothesis 3, we tested whether increases in attention were moderated by baseline levels of trait mindfulness. As seen in Table 4, the interaction between trait mindfulness, condition (Headspace), and week was significant for attention (*t*(6108) = −2.48, *p* = 0.013) at Week 8 compared to baseline. This interaction term is visualised in two panels in Figure 2, displaying the effect of week (*x*‐axis) predicting attention (*y*‐axis) at varying levels of trait mindfulness (sub‐panels). As shown in panel 2a, for the control group, as trait mindfulness increases (moving from left to right) there is little change in attention. In contrast, as shown in panel 2b, for the Headspace group, there are significant increases in attention for all levels of trait mindfulness, except those with the highest level, with steeper increases among those who were lowest in trait mindfulness. Two other patterns emerged. First, the overall effect diminished as one increased in baseline trait mindfulness (despite still showing a significant increase at all except the highest levels). Second, at baseline, those highest in trait mindfulness already showed elevated attention levels, suggesting a floor effect for potential improvement.

**TABLE 4:** Interactive effects of condition, time (in weeks), and trait mindfulness on attention and acceptance.

	Attention	Acceptance
	*b* (SE)	*b* (SE)
Random Effects		
Intercept	1.42* (0.19)	1.44* (0.19)
Residual	2.37* (0.04)	2.43* (0.04)
Fixed Effects		
Intercept	7.05* (0.76)	7.32* (0.76)
Female	0.23 (0.25)	0.23 (0.25)
Age	0.01 (0.01)	0.01 (0.01)
Hispanic	−0.10 (0.25)	0.02 (0.25)
Stress	−0.55* (0.20)	−0.60* (0.20)
Weekend	0.24* (0.05)	0.27* (0.05)
Study day	0.04* (0.02)	0.04* (0.02)
Time of day	0.00 (0.01)	0.01 (0.01)
Mindfulness	0.22 (0.24)	0.17 (0.24)
Week 2	−0.28* (0.09)	−0.28* (0.09)
Week 5	−0.35* (0.10)	−0.30* (0.10)
Week 8	−0.15 (0.11)	−0.10 (0.11)
Mindfulness × week 2	−0.00 (0.11)	0.06 (0.11)
Mindfulness × week 5	0.08 (0.12)	0.11 (0.12)
Mindfulness × week 8	0.11 (0.14)	−0.07 (0.15)
Headspace	−0.10 (0.24)	−0.13 (0.24)
Mindfulness × headspace	0.11 (0.28)	−0.03 (0.28)
Headspace × week 2	0.35* (0.11)	0.53* (0.11)
Headspace × week 5	0.69* (0.12)	0.80* (0.12)
Headspace × week 8	0.40* (0.14)	0.49* (0.14)
Mindfulness × headspace × week 2	−0.20 (0.13)	−0.14 (0.14)
Mindfulness × headspace × week 5	−0.30* (0.15)	−0.17 (0.15)
Mindfulness × headspace × week 8	−0.44* (0.18)	−0.09 (0.18)
Model Statistics		
Pseudo *R* ^2^	0.06	0.06

*Note:* Weeks 2, 5, and 8 are compared to Week 0 (baseline). Gender, Age, and Ethnicity are demographic variables and standard controls in previous tests of mindfulness‐based interventions. Weekend, Study Day, and Time of Day are standard controls in EMA studies to account for shifts that will naturally occur over time rather than due to the intervention.

Abbreviations: *b* = unstandardised beta coefficient; SE = standard error of unstandardised beta coefficient.

^*^ *p* < 0.05.

We also tested whether increases in acceptance were moderated by baseline trait mindfulness. As seen in Table 4, the interaction between trait mindfulness, being in the Headspace group, and week was not significant for acceptance (*t*(6094) = −0.48, *p* = 0.63) at Week 8 compared to baseline.

### 3.4 Perceived Stress Moderation Analyses

**FIGURE 3:**
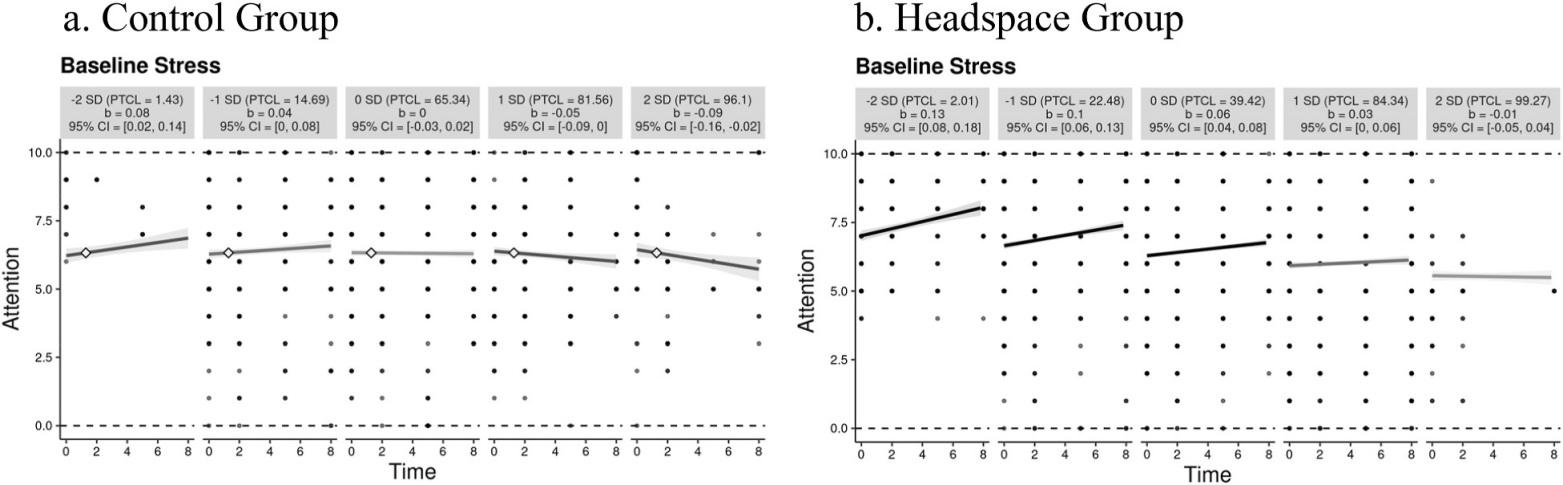
Levels of attention as a function of time (in Weeks) and perceived stress for the control (Panel 3a) and headspace (Panel 3b) conditions. (Panel 3a) Control group. (Panel 3b) Headspace group. Figure depicts the relationship between time (in weeks) and attention. (Panel A) presents the data for the control group and Panel B for the Headspace group. Within each panel, each figure is separated into five sub‐panels to visualise the effect of perceived stress as a moderator. These sub‐panels present the simple slopes for different levels of the moderator, including 2 and 1 SD below the mean, at the mean, and 1 and 2 SD above the mean. Each box above the sub‐panel graph indicates the estimated beta coefficient between time (in weeks) and attention, as well as the 95% confidence interval for the effect. Each sub‐panel displays a graph of the observed data points plotted (black dots), the 95% confidence region (shaded area) around the simple slope, and the maximum and minimum values of the outcome (dashed horizontal lines). Overall, the figures show that for the control group (Panel A), there is a curvilinear pattern with increases in attention for those with lower than average levels of stress, no change for those with average levels of stress, and decreases in attention for those with higher than average levels of stress. In contrast, for the Headspace group (Panel B), the change in attention is greater at lower levels of perceived stress and relatively flat at higher levels of perceived stress. CI = confidence interval; *PTCL* = percentile; SD = standard deviation.

To address hypothesis 4, we tested models similar to those for hypothesis 3 but substituting baseline levels of perceived stress as the moderator. As seen in Table 5, the interaction between perceived stress, condition (Headspace), and week was significant for attention (*t*(6108) = 4.03, *p* < 0.001) at Week 8 compared to baseline. This interaction term is visualised in two panels in Figure 3, displaying the effect of week (*x*‐axis) predicting attention (*y*‐axis) at varying levels of perceived stress (sub‐panels). As shown in panel 3a, for the control group, there is a curvilinear pattern with increases in attention for those with lower than average levels of stress, no change for those with average levels of stress, and decreases in attention for those with higher than average levels of stress. As shown in panel 3b, for the Headspace group attention increased for all those who had an average level or lower than average level of stress relative to the sample, although more so for those who were lowest in perceived stress at baseline.

**TABLE 5:** Interactive effects of condition, time (in weeks), and perceived stress on attention and acceptance.

	Attention	Acceptance
	*b* (SE)	*b* (SE)
Random Effects		
Intercept	1.41* (0.18)	1.44* (0.19)
Residual	2.36* (0.04)	2.42* (0.04)
Fixed Effects		
Intercept	4.79* (0.70)	5.18* (0.71)
Female	0.19 (0.25)	0.22 (0.25)
Age	0.01 (0.01)	0.01 (0.01)
Hispanic	−0.09 (0.25)	0.02 (0.25)
Mindfulness	0.21 (0.13)	0.13 (0.14)
Weekend	0.25* (0.05)	0.28* (0.05)
Study day	0.03* (0.02)	0.04* (0.02)
Time of day	0.00 (0.01)	0.01 (0.01)
Stress	−0.03 (0.37)	−0.34 (0.38)
Week 2	−0.31* (0.10)	−0.28* (0.10)
Week 5	−0.41* (0.11)	−0.34* (0.11)
Week 8	−0.39* (0.12)	−0.37* (0.12)
Stress × week 2	−0.30^a^ (0.18)	−0.10 (0.18)
Stress × week 5	−0.46* (0.21)	−0.37^a^ (0.22)
Stress × week 8	−1.17* (0.24)	−1.02* (0.24)
Headspace	−0.15 (0.24)	−0.17 (0.24)
Stress × headspace	−0.55 (0.43)	−0.13 (0.44)
Headspace × week 2	0.38* (0.11)	0.54* (0.11)
Headspace × week 5	0.74* (0.13)	0.86* (0.13)
Headspace × week 8	0.63* (0.15)	0.78* (0.15)
Stress × headspace × week 2	0.23 (0.21)	−0.09 (0.21)
Stress × headspace × week 5	0.27 (0.25)	0.06 (0.25)
Stress × headspace × week 8	1.11* (0.28)	0.71* (0.27)
Model Statistics		
Pseudo *R* ^2^	0.07	0.06

*Note:* Weeks 2, 5, and 8 are compared to Week 0 (baseline). Gender, Age, and Ethnicity are demographic variables and standard controls in previous tests of mindfulness‐based interventions. Weekend, Study Day, and Time of Day are standard controls in EMA studies to account for shifts that will naturally occur over time rather than due to the intervention.

Abbrevviations: *b* = unstandardised beta coefficient; SE = standard error of unstandardised beta coefficient.

^a^ *p* < 0.10.

^*^ *p* < 0.05.

**FIGURE 4:**
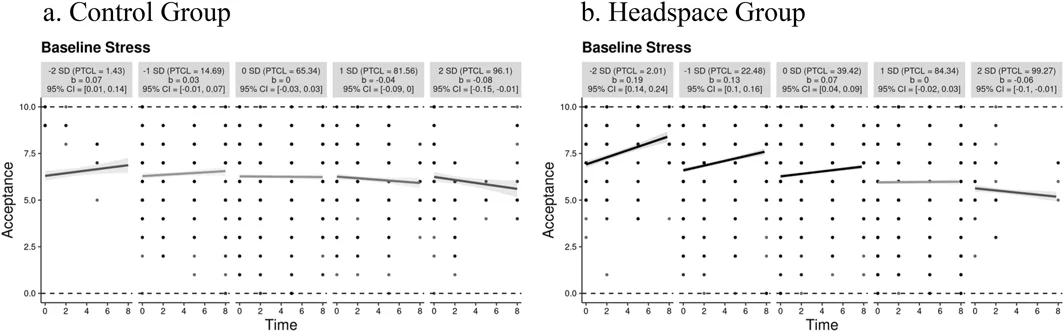
Levels of acceptance as a function of time (in Weeks) and perceived stress for the control (Panel 4a) and headspace (Panel 4b) conditions. (Panel 4a) Control group. (Panel 4b) Headspace group. Figure depicts the relationship between time (in weeks) and acceptance. (Panel A) presents the data for the control group and Panel B for the Headspace group. Within each panel, each figure is separated into five sub‐panels to visualise the effect of perceived stress as a moderator. These sub‐panels present the simple slopes for different levels of the moderator, including 2 and 1 SD below the mean, at the mean, and 1 and 2 SD above the mean. Each box above the sub‐panel graph indicates the estimated beta coefficient between time (in weeks) and acceptance, as well as the 95% confidence interval for the effect. Each sub‐panel displays a graph of the observed data points plotted (black dots), the 95% confidence region (shaded area) around the simple slope, and the maximum and minimum values of the outcome (dashed horizontal lines). Overall, the figures show that for the control group (Panel A), there is a curvilinear pattern with increases in acceptance for those with the lowest levels of stress, no change for those with 1 SD and average levels of stress, and decreases in acceptance for those with higher than average stress levels. For the headspace group (Panel B), the change in acceptance is greater at lower levels of perceived stress and relatively flat or decreasing for those with higher than average levels of perceived stress. CI = confidence interval; *PTCL* = percentile; SD = standard deviation.

We also tested whether increases in acceptance were moderated by baseline perceived stress. As seen in Table 5, the interaction between perceived stress, condition (Headspace), and week was significant for acceptance (*t*(6094) = 2.54, *p* = 0.011) at Week 8 compared to baseline. Similar to the interaction term for attention, the interaction term for acceptance is visualised in two panels in Figure 4, which display the effect of week (*x*‐axis) predicting acceptance (*y*‐axis) at varying levels of perceived stress (sub‐panels). Panel 4a shows for the control group there is a curvilinear pattern with increases in acceptance for those with the lowest levels of stress, no change for those with 1 SD and average levels of stress, and decreases in acceptance for those with higher than average stress levels. Panel 4b shows for the Headspace group acceptance increased for those with average and lower than average levels of perceived stress, although more so for those 1 or 2 SD below the average level, and it was relatively flat or decreasing for those with higher than average levels of perceived stress.

## 4 Discussion

The goal of the present study was to examine the trajectory of change for attention and acceptance skills for novice meditators while completing a mindfulness‐based intervention via the Headspace smartphone app. A further goal was to test the moderating effects of trait mindfulness and perceived stress on the trajectories of change for these key skills. Consistent with hypothesis 1, we observed statistically significant increases in attention and acceptance by Week 8 relative to Week 0 among participants assigned to the Headspace group but not those assigned to the control group. These findings are in line with conclusions of existing research indicating mHealth mindfulness‐based interventions can develop mindfulness skills (Walsh et al. 2019).

Partially consistent with hypothesis 2, among participants assigned to the Headspace group, we observed a significant increase in attention at Week 2 through Week 5, but also an unexpected significant increase in acceptance over the same period. This pattern diverges from the order of skill development described in MAT, which suggests that during mindfulness training initial practice and development of a nonjudgmental approach may follow practice and development of attention monitoring (Lindsay and Creswell 2017). It is possible that participants in this study acquired the skill of attention very quickly, and already moved on to acquiring acceptance by Week 2. In fact, previous research found the attention subscale of a mindfulness measure displayed change from week 1 to week 2 of the intervention, while change for the nonjudgement subscale was observed between weeks 1 and 3 (Baer et al. 2012). Alternatively, it may be that each skill has multiple components changing at varying paces. For example, the skill of acceptance is commonly operationalised as consisting of nonjudgement, openness and receptivity, and equanimity (Bishop et al. 2004). Prior research indicates that mindfulness subscales measuring each acceptance component display change at different weeks throughout the intervention period (Baer et al. 2012), so it may be that the single items used here were insufficient to detect skill change accurately and future tests of mindfulness skills change may need to consider including the various components. Finally, unlike in‐person mindfulness meditation interventions that commonly stagger teaching of attention and acceptance, Headspace seems to introduce these skills simultaneously in the Basics packs. This may explain the increase in both skills at Week 2, and why our findings differed from the findings of the study by Baer et al. (2012) which used in‐person training. This may imply that the simultaneous versus staggered teaching of skills is important to consider in tests of app‐based mindfulness interventions. Even so, our findings suggest those who are considered novice meditators can acquire these skills fairly quickly during participation.

In addition, our findings from our hypothesis 2 follow‐up analyses regarding the rate of change following Week 2 advance prior knowledge by indicating both key skills may not simply be the end product of participating in an app‐based mindfulness intervention, but instead are acquired and change over the course of intervention period. We found attention and acceptance increased from Weeks 0 to 2 and 2 to 5 (marginally), but decreased slightly from Weeks 5 to 8 while remaining greater than baseline levels. The increases from Weeks 0 to 2 and 2 to 5 are similar to weekly change of mindfulness found in previous research (e.g., Baer et al. 2012), although the decrease from Weeks 5 to 8 differs from findings of this previous study. Our findings display the dynamic nature of learning these key skills. Specifically, there is initial growth from Weeks 0 to 2 potentially due to high interest and perceived need, then continued learning with presumed greater practice from Weeks 2 to 5, and finally the potential for regression from Weeks 5 to 8 possibly due to fatigue. Participants may have also independently perceived a decrease (or ‘improvement’) in outcomes that served as initial motivation for volunteering for the study (e.g., high stress, high burnout) and this decrease possibly encouraged disengagement. In addition, this decrease from Week 5 to 8 may be attributed to ceiling effects, such that there may be a limit to how much more the attention and acceptance skills can increase via intervention participation. Our findings may generally demonstrate different instructions are needed throughout the intervention period and acknowledge learning these skills may not be steady.

Hypothesis 3 regarding trait mindfulness was also partially supported as we found trait mindfulness moderates the trajectory of change of the attention skill but not the acceptance skill. We had surmised higher trait mindfulness prior to the intervention would provide participants with a latent capacity advantaging them in the subsequent acquisition of the attention and acceptance skills during training, consistent with previous findings related to mindfulness (e.g., Shapiro et al. 2011). However, the confinement of the effect to change in the attention skill alone may suggest this disposition possibly confers greater attention tendencies (e.g., awareness and monitoring attention) than acceptance tendencies (e.g., nonjudgment and nonreactivity) to people who possess it. In other words, those higher in trait mindfulness may have already possessed a proclivity for attention leaving minimal room for development of this skill, while novices low on trait mindfulness were potentially a group with the most room for development of this skill. This interpretation is based on previous research indicating novice meditators experienced greater improvements on trait mindfulness from baseline to post‐training compared to regular meditators (e.g., Ito et al. 2022). It may also be the case that this finding should have been expected given that the MAAS purports to measure mainly attention. Using an attention‐focused trait measure to predict the attention skill change may have inflated observed effects. Relatedly, the tendencies conferred by trait mindfulness may be less relevant to the development of the acceptance skill, explaining why no moderating effect was observed for this specific skill. These moderation findings may have theoretical implications for understanding the relationship between trait mindfulness and the key skills of attention and acceptance.

Finally, regarding our competing predictions for hypothesis 4, we found that perceived stress moderates the trajectory of change of both the attention skill and acceptance skill with this change being greater for those lower in perceived stress rather than higher. We initially proposed competing hypotheses given previous findings of higher perceived stress moderating the effect of a mindfulness acceptance induction on coping approaches (Donald and Atkins 2016), and also research indicating higher levels of perceived stress can negatively impact learning outcomes in a different domain (Oducado and Estoque 2021). We found the moderation effect was strongest for those lower in perceived stress, which differed from previous research findings for stress management interventions. The effect of a web‐based stress management intervention on outcomes was moderated by distress, such that the intervention was more effective for those with higher distress at baseline (Coudray et al. 2019). However, previous research on general learning has displayed high levels of stress can negatively impact learning outcomes, which may have been the case in the current study. For example, the novice meditators who had higher levels of perceived stress at baseline may have been overwhelmed by the combination of high stress and attempting to learn new skills. This may explain why those who were higher in perceived stress displayed little or negative change in the mindfulness skills, while those lower displayed an increase in both attention and acceptance, although more research considering engagement is needed to support this idea. Recognising the moderating effects of perceived stress on these key skills is important to theoretical understandings of the relationship between increased mindfulness and reduced stress. For example, it is important to note that these high stress individuals may benefit from the intervention in regard to reducing stress despite not showing change in these two key skills, and future research should consider effects on treatment outcomes.

### 4.1 Practical Implications

The findings of the current study have salient implications for the use and development of mHealth apps like Headspace. Specifically, findings suggest that practicing mindfulness meditation through the Headspace app may be beneficial for people who are new to, or unfamiliar with, mindfulness meditation, in the sense that results seem to suggest people can acquire the key skills. The likelihood of skill acquisition via mindfulness apps for novice meditators is an important finding given that, to date, there has been limited indication of whether regular prior (group or independent) meditation practice is necessary to build key mindfulness skills through independent app‐based meditation practice. The findings here suggest that even those with little or no meditation experience can acquire these skills through the app‐based practice. Our findings contribute to current knowledge by highlighting the value of mHealth mindfulness interventions for acquiring mindfulness skills and particularly for novices without extensive mindfulness meditation experience (Messner et al. 2019).

Our results also suggest level of trait mindfulness and perceived stress is important when it comes to acquiring skills from participating in an mHealth mindfulness meditation intervention. As such, those interested in maximising benefits, and also in developing additional intervention components that may address individual needs, would do well to assess levels of trait mindfulness and perceived stress in advance. This requires screening participants prior to engagement in the intervention. While there is little evidence to suggest clinicians currently routinely screen for trait mindfulness and perceived stress prior to recommending mindfulness meditation, the practice of screening for potentially influential individual characteristics to facilitate personalisation of interventions has been conducted in other domains. For example, in the language acquisition domain, it is common to assess current language skills prior to assigning an individual to content that is relative to their level of proficiency (Long et al. 2018). One option may be to administer a pre‐assessment of these individual characteristics in the Headspace app when users initially sign up to enable allocation of content matched to the level of trait mindfulness and perceived stress of each individual. Future research may consider drawing focus to establishing specific cutoffs on common measures of individual characteristics, such as the PSS for perceived stress.

In addition, those with lower trait mindfulness and higher stress having less learning implies the potential need to personalise the content of mHealth apps, such as Headspace, to account for individual differences among users. Such personalisation features may include introducing new topics earlier or allowing for selection of certain content to use on specific days. Those lower in trait mindfulness may benefit from engaging in the foundational packs, such as the Basics packs in Headspace, which introduce the key skills and initiate general practice to acquire skills. While users higher in trait mindfulness may benefit from the option to access more advanced or targeted packs early on, such as Basics 3 and the Stress pack, as these individuals may already possess basic knowledge of key skills and are potentially equipped to apply the knowledge in specific contexts. Further, those higher in stress may benefit from more structure during practice and supplemental information, such as guidance and testimonials from others who have successfully used the app to reduce feelings of stress, as these individuals may be particularly overwhelmed and find it difficult to justify adding this practice to their routine. One example of personalisation is in a study testing the feasibility and efficacy of an acceptance and commitment therapy app that provided skill suggestions to participants after completing a daily check‐in (Levin et al. 2017). Incorporating personalisation features may strengthen the app so it serves a variety of users and potentially encourages engagement. Relatedly, it may be important for future investigations to test perceived stress as a moderator of the effects of app‐based mindfulness meditation on stress‐related treatment outcomes (e.g., perceived stress, burnout) as findings may clarify whether high stress individuals should be directed to alternative treatments.

### 4.2 Limitations

Findings of the current study should also be considered in light of limitations. First, our measure of trait mindfulness tended to focus on the attention component of mindfulness rather than both key mindfulness skills. This may explain why we observed a moderation effect for the attention skill only. Future studies may consider adopting mindfulness measures that encompass multiple components of the construct, such as the Five Facet Mindfulness Questionnaire which measures separate facets. This may provide a fairer test of the effects of trait mindfulness as a moderator of mindfulness skill acquisition through mindfulness apps. The FFMQ also captures other proposed components of acceptance (i.e., nonjudgment, openness and receptivity, and equanimity), which may be important to measuring change in the acceptance skill as discussed for findings of hypothesis 2. This alternative measure has been used in previous work exploring mindfulness skill change conducted by Baer et al. (2012) to measure skills individually and mindfulness more generally. Identifying if there are differences in effects according to the trait mindfulness measure used would also be informative for practitioners who may want to assess mindfulness with an established measure prior to assigning the intervention.

In addition, the psychometric properties of the EMA items used to measure the attention and acceptance skills are important to consider. The items used were adapted from an established mindfulness measure, but validity and reliability of these items has not been formally tested. Data collection may have been limited by the use of single items (i.e., one for each skill). In supplementary analyses examining correlations between the baseline MAAS and the single‐item EMA measures for each skill, significant weak correlations were found for both attention (*r*(6262) = 0.15, *p* < 0.001) and acceptance (*r*(6248) = 0.12, *p* < 0.001). Thus, the findings of this study should be interpreted with this in mind, and future research should focus on creating validated measures of items assessing mindfulness skills for use in EMA research. Relatedly, future research should consider whether presenting EMA items measuring attention and acceptance skills in a different order each time throughout data collection is important to prevent order effects.

Importantly, we did not have access to continuous usage (or engagement) data for Headspace. The study had originally intended to collect end‐of‐day reports regarding use of Headspace, but there were technical errors in which participants were not prompted to report this information. This constrained our ability to test whether loss of engagement helped to explain the differences in skill acquisition across moderator levels. It is important for future investigations to test engagement in relation to moderators, such as perceived stress and mindfulness, to clarify if levels of moderators influence engagement in the Headspace meditation practices. Particularly for the high perceived stress group, this may be useful in determining whether this group has low engagement levels throughout the intervention period to further clarify the findings of the present study regarding low skill acquisition in the group.

We also note substantive participant attrition pre‐to post‐intervention. A high rate of attrition is not uncommon for app‐based mindfulness intervention studies (Linardon 2023), and baseline levels of mindfulness or stress and demographic characteristics did not differ between completers and non‐completers. However, there may have been other undetected differences between these groups. The commonality of a high attrition rate indicates the need to focus on identifying *why* dropout tends to be so high in studies testing the effects of these interventions. It is important to pinpoint whether this is a result of individual differences, a product of barriers to participation, the structure of the intervention itself, or a combination of factors. Future research should focus on distinguishing reasons for dropout and proposing methods that can potentially be employed in these studies to promote sustained participation.

Finally, despite the use of an initial screening survey, it is important to acknowledge the possibility of self‐selection bias limiting generalisability and that findings may not generalise to clinical populations.

## 5 Conclusion

Our findings corroborate those of previous studies indicating mHealth mindfulness apps can increase mindfulness. Novice meditators who used the Headspace app showed change in mindfulness skills by the end of the intervention period and the key skills of attention and acceptance displayed change across the intervention period with initial change occurring as early as 2 weeks. Our findings also indicate that those with lower trait mindfulness at the beginning of the intervention period show greater change in attention and those with lower perceived stress at the beginning show greater change in both attention and acceptance. This suggests it may be important to assess characteristics prior and potentially expand personalisation features. There remains a need to consider the appropriateness of tools used to measure trait mindfulness and to address high attrition rates that are common in mindfulness‐based intervention studies.

## Conflicts of Interest

The authors declare no conflicts of interest.

## Data Availability

The original study was registered with ClinicalTrials.gov (NCT03652168) and the current study was preregistered on the OSF repository (https://doi.org/10.17605/OSF.IO/8TGX7). Data is not available in a repository as participants did not consent to data sharing, but is available on request pending appropriate data sharing agreements.
